# Lynch syndrome: from detection to treatment

**DOI:** 10.3389/fonc.2023.1166238

**Published:** 2023-05-01

**Authors:** Madeleine H. Williams, Andreas V. Hadjinicolaou, Benjamin C. Norton, Rawen Kader, Laurence B. Lovat

**Affiliations:** ^1^ Department of Gastroenterology, Guy’s and St. Thomas NHS Foundation Trust, London, United Kingdom; ^2^ Department of Gastroenterology, Cambridge University Hospitals NHS Foundation Trust, Cambridge, United Kingdom; ^3^ Early Cancer Institute, Department of Oncology, University of Cambridge, Cambridge, United Kingdom; ^4^ Department of Gastroenterology, University College London Hospitals NHS Foundation Trust, London, United Kingdom; ^5^ Wellcome-EPSRC Centre for Interventional and Surgical Sciences, University College London, London, United Kingdom

**Keywords:** lynch syndrome, mismatch repair (MMR) deficiency, colorectal cancer, surveillance, cancer diagnosis, cancer treatment

## Abstract

Lynch syndrome (LS) is an inherited cancer predisposition syndrome associated with high lifetime risk of developing tumours, most notably colorectal and endometrial. It arises in the context of pathogenic germline variants in one of the mismatch repair genes, that are necessary to maintain genomic stability. LS remains underdiagnosed in the population despite national recommendations for empirical testing in all new colorectal and endometrial cancer cases. There are now well-established colorectal cancer surveillance programmes, but the high rate of interval cancers identified, coupled with a paucity of high-quality evidence for extra-colonic cancer surveillance, means there is still much that can be achieved in diagnosis, risk-stratification and management. The widespread adoption of preventative pharmacological measures is on the horizon and there are exciting advances in the role of immunotherapy and anti-cancer vaccines for treatment of these highly immunogenic LS-associated tumours. In this review, we explore the current landscape and future perspectives for the identification, risk stratification and optimised management of LS with a focus on the gastrointestinal system. We highlight the current guidelines on diagnosis, surveillance, prevention and treatment and link molecular disease mechanisms to clinical practice recommendations.

## 1 Introduction

Lynch Syndrome (LS) is a hereditary cancer predisposition syndrome characterised by a high lifetime risk of developing cancers, primarily colorectal and endometrial (1). These cancers exhibit microsatellite instability (MSI) due to defects in the cellular mismatch repair (MMR) system (2). LS is associated with other malignancies including gastrointestinal (GI) (e.g. gastric, small intestinal, hepato-biliary and pancreatic) and extra-GI cancers (e.g. prostate, ovaries, skin, central nervous system and upper urinary tract) (3). LS follows an autosomal dominant pattern of inheritance with germline pathogenic variants in one of the MMR genes, which, in health, maintain genomic stability (4). An estimated 1/450 people in the UK have LS (5), and of those, only 5% are diagnosed. The lifetime risk of colorectal cancer (CRC) in LS patients can vary from 10-80% dependent on the MMR mutation and age, and it is thought to be responsible for 3-5% of all CRCs (6, 7). This makes LS one of the most frequently encountered cancer susceptibility syndromes.

A prototypical cancer surveillance programme using colonoscopy exists for CRC in the setting of LS, but quality data on the role of surveillance for other LS-associated tumours is limited. In recognition of the growing need for new approaches to improve survival, this review explores the current landscape and future perspectives for the detection, risk stratification and management of LS.

## 2 Identification

### 2.1 LS genetics

LS is due to a pathogenic variant within one of the MMR genes: *MLH1*, *MSH2*, *MSH6* or *PMS2* (4). *MLH1*/*MSH2* mutations are responsible for 70-90% of LS cases and carry significantly higher lifetime cancer risk (8). A small proportion of LS cases (1-3%) arise secondary to constitutional epimutations of the *MLH1* or *MSH2* genes (9). The heterozygous, loss-of-function, germline mutations in MMR genes are phenotypically dominant but may also convey vulnerability to a second, somatic mutation in the wildtype (normal) allele. Tumorigenesis then develops due to deficient mismatch repair (dMMR) and accumulation of further mutations including in small regions of repeated DNA called microsatellites. This gives rise to microsatellite instability (MSI); the genetic signature of LS-associated tumours.

The need to differentiate between sporadic and inherited CRC in patients with dMMR tumours is crucial because of downstream implications for cancer surveillance. Unfortunately, this is not always straightforward and we are increasingly aware of a heterogenous patient group with Lynch-like syndrome (LLS) defined as dMMR tumours where LS is suspected but no pathological germline MMR mutation is identified (10).

### 2.2 Diagnosis

The diagnosis of LS is made in symptomatic patients presenting with a LS-associated cancer, or among asymptomatic patients with a confirmed familial pathogenic variant. In symptomatic cases, the tumour is subjected to molecular profiling for evidence of dMMR. MSI is assessed either using polymerase chain reaction (PCR)-based testing or loss of/abnormal protein expression of *MLH1*, *MSH2*, *MSH6* or *PMS2* using immunohistochemistry (IHC) (11). Both methods have high sensitivity (PCR 92.9%, IHC 92.4%), specificity (PCR 86.3%, HCI 87.8%) and negative predictive values (PCR 99.6%, IHC 99.6%) for LS (12).

An abnormal result must be followed by referral for genetic testing and counselling. Younger patients (<40 years old) should be referred directly for germline testing according to the NHS National Genomic Medicine Service (GMS) Lynch Syndrome Project guidelines (13). Among families with a confirmed pathogenic MMR variant, asymptomatic patients can be referred for cascade genetic testing directly without the need for findings consistent with CRC.

Since 2017, the National Institute for Clinical Excellence (NICE) has recommended testing all newly identified CRCs for dMMR by IHC or for MSI to guide the need for LS evaluation (11). This guidance was expanded to IHC testing in all new endometrial cancers in 2020 (14). These recommendations have superseded the previously used Amsterdam Criteria and Bethesda Guidelines (15, 16) which mainly relied on crude measures such as family history and age of cancer onset (17). Looking to the future, NICE have proposed an accelerated review of next generation sequencing (NGS) as a potential index test for paired tumour-germline profiling in all newly diagnosed CRCs (18). NGS enables identification of MSI using computational algorithms such as mSINGS, MSISenory, and MANTIS among others (19). It can simultaneously sequence the whole exome looking for markers of MSI, compared to a normal/baseline sample, which is measured against a threshold value. Concurrently, exome tumour sequencing can be paired with a blood sample to enable differentiation between somatic and germline variants (20). This paired testing is superior to traditional stepwise testing, which would enable earlier, more precise and personalised risk stratification in suspected LS cases (21).

### 2.3 Determining cancer risk

Over the last few decades, there has been great insight into the natural history of LS patients with thousands of unique germline MMR gene variants identified and recorded in international databases such as InSiGHT (22). However, having a pathogenic variant does not result in a uniform diagnosis across all patients, with great genetic variability observed due to penetrance (i.e. the probability of a gene being expressed) and expressivity (i.e. if the gene is penetrant, the variability in that expression). The establishment of the Prospective Lynch Syndrome Database (PLSD), an international, multi-centre, observational prospective study, has improved understanding of the cumulative incidence and survival of LS-associated cancer patients (between 25-75 years) and equipped us with age and cancer-specific risk estimates for each pathogenic MMR variant (**Table 1**) (24, 25). However, it is important to acknowledge its limitations such as the absence of a control group who did not undergo surveillance and granular data such as cancer-specific survival.

**Table 1:** Cumulative incidence of individual cancers in patients with pathogenic MMR variants between 25-75 years old (23).

Cancer type		Cumulative cancer risk at age 75 years (% (95% CI))			
		*path_MLH1*	*path_MSH2*	*path_MSH6*	*path_PMS2*
**Colorectal**	Colon	46.7 (39.2 to 54.3)	42.4 (32.9 to 51.9)	14.2 (3.1 to 25.4)	0
	Sigmoid and rectum	11.8 (7.2 to 16.4)	18.3 (10.9 to 25.6)	4.6 (0.0 to 9.7)	0
**Gynaecological**	Endometrium	42.7 (33.1 to 52.3)	56.7 (41.8 to 71.6)	46.2 (27.3 to 65.0)	26.4 (0.8 to 51.9)
	Ovaries	10.1 (4.8 to 15.4)	16.9 (5.7 to 28.0)	13.1 (0.0 to 31.2)	0
**Upper GI**	Stomach	7.1 (3.5 to 10.8)	7.7 (1.9 to 13.6)	5.3 (0.0 to 13.1)	0
	Duodenum	6.5 (2.7 to 10.2)	2.0 (0.1 to 4.0)	0	0
	Biliary	3.7 (1.3 to 6.2)	1.7 (0.0 to 5.1)	0	0
	Pancreas	6.2 (2.6 to 9.8)	0.5 (0.0 to 1.5)	1.4 (0.0 to 4.2)	0
**Urinary tract**	Bladder	4.1 (1.5 to 6.7)	8.1 (2.8 to 13.3)	8.2 (0.0 to 16.9)	0
	Kidneys and ureters	4.6 (1.6 to 7.6)	17.8 (10.6 to 25.0)	3.0 (0.0 to 7.0)	0
**Other**	Brain	1.0 (0.0 to 2.4)	5.3 (0.2 to 10.3)	1.4 (0.0 to 4.2)	0
	Prostate	16.9 (8.5 to 25.3)	31.6 (11.7 to 51.5)	18.3 (0.0 to 44.4)	37.9 (0.0 to 95.9)
	Breast	12.0 (6.7 to 17.3)	11.5 (4.6 to 18.4)	13.3 (2.2 to 24.4)	55.9 (0.0 to 100.0)

These limitations have somewhat been addressed by the international multi-centre International Mismatch Repair Consortium (IMRC) (26). In contrast to the PLSD, in which all cases have undergone at least one colonscopy, IMRC data derives from retrospective segregation analysis of LS families, including older generations who did not receive comparable colonoscopic surveillance. Contrary to expectations, incidence of CRC in *path_MLH1* and *path_MSH2* carriers in the PLSD group (who underwent colonoscopy and polypectomy) was significantly higher than in the IMRC series. Differences in data fidelity between the two databases could have influenced these findings (27).

## 3 Risk stratification

Over the last decade, significant improvements have been made in the personalised risk stratification of patients with LS. However, the optimal timing of surveillance is still to be determined and there is a paucity of data for extra-colonic tumours and surveillance in older age patients (28).

### 3.1 Colorectal cancer surveillance

Current consensus favours conoloscopy for CRC surveillance in asymptomatic patients with LS. A landmark prospective study from Finland in 2000 demonstrated that 3-yearly colonoscopy in LS decreased CRC incidence and mortality (29, 30), with other non-randomised studies replicating these findings (31, 32). However, many of these are somewhat limited in their granularity of data. For example, in the aforementioned study, all participants who attended a colonscopy were deemed to be compliant with surveillance regardless of the frequency of their surveillance or whether they had any actual further colonoscopies at all. More recently, a retrospective cohort study (33) used a unique time-based model to explore the effect of surveillance interval in LS (<27 months vs >27 months vs no surveillance), demonstrating that shorter intervals reduced the risk of first CRC diagnosis. These findings could encourage adherence to timely surveillance in at-risk individuals, although an important limation of this study in the context of colonoscopy, was the inclusion of other surveillance techniques such as CT colonography, MRI and barium enema.

The optimal strategy for CRC surveillance in LS remains the subject of ongoing research. Guidelines vary internationally (outlined in **Table 2**) (3, 10, 34–45), with the European Society of Gastrointestinal Endoscopy (ESGE) recommending 2 yearly (36). Interestingly, 98% of centres favoured colonscopy every 1-2 years when reported to the IMRC (46). The prevalence of CRC is low in patients with LS under the age of 25 regardless of genotype, however data from both PLSD and IMRC support the notion that those with the higher penetrance *MHL1* and *MSH2* variants typically develop CRC earlier in life than their *MSH6* and *PMS2* counterparts (26, 47), hence the decision by some to begin surveillance earlier for *MSH1/MSH2* carriers (**Table 2**). In patients with *PMS2* variants, carcinogenesis may be more akin to the traditional adenoma-carcinoma sequence (25, 48) leading to low CRC incidence which may justify the suggestion from the European Mallorca guidelines for 5-yearly surveillance (35).

**Table 2:** Current recommendations for colorectal cancer surveillance from different national and international organisations.

Country/Continent of origin	Organisation	Age to start surveillance (with corresponding pathological MMR gene mutation, where applicable)	Surveillance interval (with corresponding MMR gene mutation, where applicable)	Comments
**Australia**	Cancer Institute of New South Wales (34)	25 years (*MLH1/MSH2)* 35 years (*MSH6/PMS2*)	1-2 years	Review all cases at age 60 years with a view to reducing frequency
**Europe**	Mallorca Guidelines from The European Hereditary Tumour Group (EHTG) and European Society of Coloproctology (ESCP) (35)	25 years (*MLH1/MSH2)* 35 years (*MSH6/PMS2*)	2-3 years (*MLH1/MSH2/MSH6)* 5 years (*PSM2*)	
	European Society of Gastrointestinal Endoscopy (ESGE) Guideline (36)	25 years (*MLH1/MSH2)* 35 years (*MSH6/PMS2*)	2 years	
	European Society of Medicine (ESMO) (37)	25 years (*MLH1/MSH2)* 35 years (*MSH6/PMS2*)	1-2 years	Offer colonoscopy 5 years younger than age of youngest diagnosed CRC case in family (if diagnosed before age 25)
**France**	French National Authority for Health (38)	25 years	2 years	Offer colonoscopy 5 years younger than age of youngest diagnosed CRC case in family (if diagnosed before age 25)
**Germany**	German Consortium for Familial Colorectal Cancer (39)	25 years	1-2 years	
**Japan**	Japanese Society for Cancer of the Colon and Rectum (JSCCR) (40)	20-25 years	1-2 years	
**Netherlands**	Integrated Cancer Centre Netherlands (41)	25 years	2 years	
**Spain**	Spanish Society of Medical Oncology (SEOM) (42)	20-25 years	1-2 years	Offer colonoscopy 2-5 years younger than age of youngest diagnosed CRC case in family (if diagnosed before age 25)
**UK**	British Society of Gastroenterology BSG)/Association of Coloproctology of Great Britain and Ireland (ACPGBI)/United Kingdom Cancer Genetics Group (UKCGG) (10)	25 years (*MLH1/MSH2*) 35 years (*MSH6/PMS2*)	2 years	Until age 75 years
**USA**	US Multi-Society Task Force (USMSTF) (43)	20-25 years *(MLH1/MSH2)* 30 years (*MSH6*) 35 years (*PMS2*)	1 year	Offer colonoscopy 2-5 years younger than age of youngest diagnosed CRC case in family (if diagnosed before age 25)
	American College of Gastroenterology (ACG) (3)	20-25 years (*MLH1/MSH2)* 25-30 years (*MSH6/PMS2*)	1-2 years	
	American Society of Clinical Oncology (ASCO) (44) ^1^	25 years (*MLH1/MSH2)* 35 years (*MSH6/PMS2*)	1-2 years	Offer colonoscopy 5 years younger than age of youngest diagnosed CRC case in family (if diagnosed before age 25)
	National Comprehensive Cancer Network (NCCN) (45)	20-25 years (MLH1/MSH2) 30-35 years (MSH6/PMS2)	1-2 years 1-3 years	Offer colonoscopy 2-5 years younger than age of youngest diagnosed CRC case in family (if diagnosed before age 25)

Whilst 1-2 yearly colonoscopy in LS is widely practiced, prospective observational cohorts have demonstrated that lifetime risk of CRC, including metchronous tumours, remains as high as 36% (49, 50) and do not necessarly improve by increasing surveillance frequency (51). Analysis of 2747 LS patients showed no significant difference between incidence and stage of CRC between annual, 1-2 yearly and 3 yearly surveillance (52). It has also been suggested that frequent surveillance could lead to over-diagnosis by detecting tumours that may not have become clinically significant (53). Compliance issues too may be an argument for longer surveillance intervals. In one study, loss to follow-up rates were higher among participants randomised to annual screening than those having 2 or 5-yearly surveillance (54). Considering these findings it is perhaps unsurprising that consensus on surveillance strategy is difficult to establish.

There are various hypotheses as to why the rate of interval CRC is still high despite best efforts in surveillance programmes. First, it has been suggested that CRC in LS develops through accelerated tumorigenesis compared with sporadic CRC (55). This assumes a prior optimally performed colonoscopy. Second, adenomas in LS are often proximal, flat, and harder to detect, which could lead to missed lesions, especially during inadequately performed colonoscopy (56). Finally, LS-associated CRCs may have a unique, non-polypous carcinogenesis pathway that allow them to develop from endoscopically undectable lesions (e.g. colonic crypts) (57). The aforementioned failure to reduce CRC incidence by reducing surveillance intervals suggests that accelerated carcinogenesis is less likely and has led to a switch of focus on optimising the colonoscopic procedure and adherence to key performance indictors for colonoscoy (10, 58–60).

#### 3.1.1 Advanced imaging and artificial intelligence

High quality colonoscopy is crucial to the detection of both sporadic and hereditary CRC (61), especially in LS where lesions may be difficult to detect. To achieve this, different advanced imaging modalities including dye-based and virtual chromoendoscopy (VCE) have been assessed in patients with LS. A recent meta-analysis of four prospective studies comparing standard white light endoscopy (WLE) to chromoendoscopy using dye-spray showed that the latter was superior for detection of any adenomatous, flat, or proximal lesion (62). European guidelines suggest chromoendoscopy as an adjunct, whereas BSG guidelines advise that it offers no advantage to high-definition white light endoscopy (HDWLE) (10, 35, 36).

VCE is increasingly popular owing to its ease of use. Back-to-back studies comparing imaging modalities immediately following one another have shown a benefit for both narrow band imaging (NBI; Olympus) and iScan (Pentax) in LS polyp detection (63, 64). However, these comparisons have also shown higher lesion detection with dye-based chromoendoscopy versus NBI (65, 66). A recent multi-centre RCT compared HDWLE to Linked colour imaging (LCI; Fujifilm) among 357 patients with pathogenic LS variants and found no significant difference in polyp detection rate (44.4% vs. 36.0%; p=0.12) (67). Thus,at best, advanced imaging techniques can be an adjunct to HDWLE but cannot replace standard care.

In another growing field, the use of real-time artificial intelligence (AI)-colonoscopy has demonstrated enhanced detection of polyps and adenomas in average risk CRCs (68–71). A recent German RCT demonstrated a higher (albeit not statisticaly significant) rate of lesion detection, including LS-relevant flat lesions, by AI-colonoscopy than HDWLE in a LS cohort (72).

#### 3.1.2 Non-invasive screening

A recent systemic review (73) brought attention to non-invasive biomarkers such as plasma-based methylated SEPTIN9, Big Adenine Tract-26 (a faceal marker of MSI), faecal sulfate-reducing bacteria *Desulfovibrio* and faecal immunochemical testing (FIT) in the detection of CRC and adenomas in LS, although further evidence is required to support their use in practice. A 2017 meta-analysis reported that FIT had a sensitivity of 85% for CRC and 46% for advanced adenomas in asymptomatic adults with a family history, suggesting that FIT alone would miss advanced neoplasia (74). However, during the COVID-19 pandemic in England, when access to non-urgent colonoscopy services was restricted, a temporary system based on FIT was introduced to risk stratify patients with LS to urgent colonoscopy (75). This formed the basis for an ongoing UK-based multi-centre prospective study examining a potential future role for FIT testing in LS (76).

### 3.2 Extra-colonic surveillance

Recommendations for the surveillance of LS-associated extra-colonic cancers are vary. For gastric cancers, most guidelines support routine testing for, and eradication of, *Helicobacter pylori*. American, Japanese and certain European guidelines advocate for regular oesophagogastroduodenoscopy (OGD) starting from 30-35 years of age (3, 37, 40, 77).

Beyond careful inspection of the duodenum and terminal ileum at OGD and colonoscopy respectively, routine testing for small bowel cancers is not typically recommended, though capsule endoscopy has been suggested for unexplained iron deficiency anaemia or abdominal pain (78).

LS families have been estimated to have an 8.6-fold increased risk of pancreatic cancer compared to the normal population (79) and surveillance using MRI or endoscopic ultrasound has been proposed for high-risk groups and carriers (80). However, low diagnostic yields and poor outcomes from surgical treatment of suspicious pancreatic lesions largely negate any theoretical benefit (10). Surveillance practices for LS-associated gynaecological cancers lack consensus and have not demonstrated a mortality benefit (81). American and European Oncology guidelines advocate for annual transvaginal ultrasound and endometrial sampling from the ages of 30-35, and prophylactic hysterectomy and bilateral salpingoophrectomy once child bearing completes, although the evidence for this is weak (3, 35, 37, 77). There is currently insufficient evidence to recommend screening for other extra-colonic LS cancers.

Unlike CRCs, for which standarised mortality ratios have been reported to decrease over time in LS cohorts, risk of death from LS-associated extra-colonic tumours is significantly increased compared with the general population (82). In a retrospective Finnish cohort, 7.2% of patients developed urothelial, prostate or gastric cancer, with one in five dying from the disease (83). Extra-colonic surveillance may benefit those with cancer at a young age who have a higher lifetime risk of subsequent cancer, but this needs addressing in well-designed prospective trials.

## 4 Management

### 4.1 Preventative interventions

#### 4.1.1 Modifiable risk factors

Most data on modifiable risk factors such as poor diet, high alcohol intake, smoking, lack of exercise and high body mass index (BMI) are extrapolated from sporadic CRC cohorts (84). Weak evidence specific to LS suggests lower CRC risk in patients who consume more fruit and higher risk in smokers (85). Subgroup analyses from the Colorectal Adenoma/Carcinoma Prevention Programme 2 (CAPP2) trial revealed a significant association between obesity and CRC risk (86). Two prospective cohort studies demonstrated a 30% increased risk of CRC for every 5.0 kg/m^2^ increase in BMI in early adulthood and an association between an overweight BMI and CRC risk in men (87, 88).

#### 4.1.2 Chemoprophylaxis

Aspirin is the only recommended chemoprophylaxis in LS. Its potential benefit was first highlighted by meta-analyses associating long-term use with lower incidence of all cancers, especially proximal CRC (89, 90). Subsequently the double-blinded RCT CAPP2, of 861 LS patients demonstrated that the use of 600mg/day of aspirin for 2-4 years was linked with a significantly lower risk of all LS-associated cancers after 10 year follow-up (91). A successive ongoing trial, CAPP3, aims to establish optimal dosing, meanwhile international guidelines have varied in their adoption of the CAPP2 findings. In the UK, both the BSG and NICE support the use of 150mg aspirin daily (300 mg if obese) in patients under 70 years old for 2-5 years (10, 92). American guidelines by contrast have refrained from recommending its use given data is currently derived from a single trial (3, 77).

### 4.2 Endoscopic and surgical management

Data on advanced endoscopic techniques to remove early-stage colorectal tumours in LS is lacking, therefore current practice heavily favours surgical resection. Endoscopic management follows guidance for non-LS colorectal polyps (93). As such, it is critical to optimise complete resection rates in LS-associated polypectomies, particularly for flat serrated polyps (94, 95).

The role of surgery in LS-associated CRC is two-fold: to resect the advanced neoplastic lesion and reduce the risk of metachronous disease. Meta-analyses have demonstrated a lower incidence of metachronous CRC in those who underwent extended resection (total/subtotal colectomy with ileorectal/ileosigmoidal anastomosis) versus segmental resection for a first CRC (96, 97) with absolute risk for metachronous tumour of 4.7% and 22.4%, respectively, over 100.7 months follow-up (98).

The risk of metachronous disease applies mainly to *MHL1* and *MSH2* pathogenic variant carriers and thus, in this context, most guidelines recommend the use of extended colectomy for a first CRC, particularly in younger patients (3, 10, 35, 37). For carriers of *MSH6* and *PMS2* variants there is insufficient evidence of oncological benefit to support the same approach, thus,for a first CRC, UK guidelines consider the two surgeries equal (10), whereas European guidelines advocate segmental resection unless there is a metachronous CRC (35).

### 4.3 Oncological management

#### 4.3.1 Chemotherapy

Systemic anti-cancer treatment options for LS-CRCs were previously confined to the four chemotherapeutic agents used in sporadic CRCs (fluorouracil, leucovorin, oxaliplatin and irinotecan) with no consideration given to MSI or MMR status. Studies that explored the efficacy of these treatments in MSI-high CRCs were conflicting, not specific to LS and limited by small sample sizes (99–102). A single LS-CRC-specific retrospective study found no survival benefit associated with adjuvant fluorouracil (103). Nevertheless these agents remain in use as adjuvant treatment for some high-risk or late stage MSI-H/dMMR CRCs, both sporadic and LS-associated (104).

#### 4.3.2 Immunotherapy

MMR-deficient CRCs demonstrate higher levels of immunogenicity than their MMR-proficient counterparts. MMR deficiency allows accumulation of point mutations in microsatellite sequences which can cause translational frameshifts, generating carboxy-terminal frameshift peptides (FSPs) that serve as “neoantigens” recognised by and stimulating the anti-tumour host immune response. The immunoreactive nature of MSI-high/dMMR CRCs prompted use of checkpoint inhibitors. The phase three KEYNOTE-177 trial demonstrated that pembrolizumab (anti-PD1) doubles the median progression-free survival compared to standard chemotherapy (16.5 vs 8.2 months) (105). As such, pembrolizumab is now approved by the USA Food and Drug Administration and recommended first-line treatment in the UK for metastatic MSI-high/dMMR CRCs. A second PD-1 inhibitor, nivolumab, is also NICE-approved for combination use with ipilimumab following standard combination chemotherapy (106).

It remains unknown whether LS-CRCs and sporadic MSI-high/dMMR CRCs share a common response to checkpoint inhibitor therapy. The higher neoantigen load in LS-CRCs might suggest an even more pronounced response, but available studies of checkpoint inhibitors that include LS patients are largely limited by small subgroup numbers and have not demonstrated a difference in response rates (107–110).

#### 4.3.3 Vaccines

The compelling evidence for interplay between host immune surveillance and LS tumours has provided the conceptual basis for the use of vaccines to augment the adaptive immune response in LS. The high burden of foreign FSPs in LS makes them excellent vaccine targets (111, 112). Although not specifically tested in LS-CRC, FSP-based vaccination induced significant humoral and T-cell responses in a first-in-human, phase I/IIa clinical trial (113) as well as in a mouse model of conditional *MSH2* knockout (114). The same principles underpin the use of cancer vaccines to prevent tumour development from premalignant polyps by targeting CRC-associated antigens such as MUC1 and CEA, a theory currently being tested and with promising results in mouse models (115, 116).

## 5 Conclusion

Lynch syndrome is encountered by many clinicians at some stage in their practice and yet remains under-diagnosed with historically limited success in risk stratification and management. The PLSD international database continues to expand our knowledge of LS-associated cancer risk. However, we have yet to obtain international consensus on the optimal surveillance strategies, which will be essential among a population of patients who are living beyond their index cancer. The advent of NGS into clinical practice will undoubtably improve detection rates and allow for more effective, precise, and personalised management programmes for patients with LS. Finally, over the next decade it will be exciting to see improvements in the preventative strategies that can be offered to patients in the form of aspirin, or even anti-cancer vaccines, as we continue to attempt to disrupt the natural history of this prevalent cancer predisposition syndrome.

## Author contributions

MW, AH, BN, RK and LL contributed to conception and design of the review. MW, AH and BN performed literature review and wrote the first draft of the manuscript. All authors contributed to the article and approved the submitted version.
